# Pyruvate is required for catecholamine-stimulated growth of different strains of *Campylobacter jejuni*

**DOI:** 10.7717/peerj.10011

**Published:** 2020-09-28

**Authors:** Meicen Liu, Mark Lyte

**Affiliations:** Department of Veterinary Microbiology and Preventive Medicine, College of Veterinary Medicine, Iowa State University, Ames, IA, United States of America

**Keywords:** *Campylobacter jejuni*, Catecholamines, Stress, Norepinephrine, Dopamine, Iron, Microbial endocrinology, Pyruvate

## Abstract

Humans and food-producing animals are constantly exposed to and affected by stress. As a consequence of stress, the release of stress-related catecholamines, such as norepinephrine (NE) and dopamine (DA), from nerve terminals in the gastrointestinal tract potentiates both the growth and the virulence of pathogenic bacteria. This may lead to the enhancement of gastrointestinal infections in humans or food-producing animals. Compared with foodborne bacterial pathogens such as *Escherichia coli* and *Salmonella* spp., less is known about the effect of stress catecholamines on *Campylobacter jejuni* subsp. *jejuni*. The present study focuses on the effect(s) of stress catecholamines DA and NE in iron-restricted media and how they affect the growth of different *C. jejuni* strains NCTC 11168, 81–176, and ML2126. Results demonstrated that DA- and NE-enhanced growth of *C. jejuni* in iron-restricted media may involve different mechanisms that cannot be explained by current understanding which relies on catecholamine-mediated iron delivery. Specifically, we found that DA-enhanced growth requires pyruvate, whereas NE-enhanced growth does not. We further report significant strain-specific dependence of *C. jejuni* growth on various catecholamines in the presence or absence of pyruvate. These data provide novel insights into the effect(s) of stress catecholamines on the in vitro growth of *C. jejuni* in iron-restricted environments, such as the intestinal tract. They suggest a mechanism by which stress-related catecholamines affect the growth of *C. jejuni* in the intestinal tract of food-producing animals, which in turn may influence colonization and transmission to humans.

## Introduction

*Campylobacter* spp. are the leading cause of foodborne bacterial disease worldwide, of which *Campylobacter jejuni* is the most frequently isolated species from patients with *Campylobacter*-associated infections (campylobacteriosis) (Havelaar et al., 2015). Although *C. jejuni* is an intestinal commensal found in a wide range of mammals and birds, human campylobacteriosis is characterized by varying clinical symptoms including fever, abdominal cramps, vomiting, mild to severe diarrhea or the immune-mediated neuropathy Guillain-Barre syndrome (Butzler, 2004; Skarp, Hänninen & Rautelin, 2016; Goodfellow & Willison, 2016). Food-producing animals such as poultry, cattle and sheep are the main reservoirs for human campylobacteriosis. The disease is mainly transmitted through consumption and handling of contaminated meat, milk, or water. Attempts to prevent *C. jejuni* colonization in food-producing animals have produced limited success (Johnson, Shank & Johnson, 2017; Wagenaar, French & Havelaar, 2013; Meunier et al., 2017; Vandeputte et al., 2019; Richards, Connerton & Connerton, 2019).

Stress is recognized as a factor contributing to the development of enteric infection, and may be crucial to colonization of *C. jejuni* in food-producing animals. Both psychological and physiological stress have been shown to alter the gut microbiome composition, resulting in the overgrowth of pathogenic bacteria in different animal hosts, including humans (Mawdsley & Rampton, 2005; Franzosa et al., 2019; Zijlmans et al., 2015), mice (Bailey et al., 2010; Dréu et al., 1999; Hendrickson et al., 1999), chickens (Shi et al., 2019), and dairy cows (Chen et al., 2018). It should be noted that food production animals are exposed to a variety of stressors such as extreme weather, confinement, high population density, and human handling (Morgan & Tromborg, 2007).

Stress may contribute to increased susceptibility to enteric bacterial diseases by increasing the release of neurochemicals from the enteric nervous system (ENS) into the intestinal lumen, especially those belonging to the catecholamine family (Lyte, 2016a). Norepinephrine (NE) and dopamine (DA) are two of the main stress-related catecholamines found in the ENS that can also be found within the gut lumen (Aneman et al., 1996; Eisenhofer et al., 1997). During periods of acute stress, NE can be released from the ENS into the gut lumen, where it can interact with the intestinal microbiota (Lyte & Bailey, 1997; Freestone, 2013). Dopamine can be released from dopaminergic nerves that constitute part of the ENS (Li et al., 2004) which are located in the mucosal layer, as well as the submucosal (Meissner) and myenteric (Auerbach) plexuses of the intestinal wall (Mittal et al., 2017; Li et al., 2006). When rats were exposed to cold-restrained stress, DA concentration increased in the colon (Zhang et al., 2012). These data suggest that neural pathways may contribute to the release of DA into the intestinal lumen during periods of stress where DA may interact with *C. jejuni*. Both NE and DA containing nerve fibers are found in the Peyer’s patches in porcine jejunum (Green et al., 2003). Contraluminal administration of NE enhanced the uptake of enteropathogenic bacteria in Peyer’s patches, a process that may contribute to the humoral response against these bacteria (Green et al., 2003). Since *C. jejuni* has been shown to selectively adhere to and translocate through Peyer’s patches (Walker et al., 1988), this finding suggests that catecholamine-containing nerve fibers may modulate the mucosal immune response and affect the outcome of *C. jejuni* infections.

The theoretical framework of microbial endocrinology has been previously employed to study the effects of stress-related neurochemicals, notably catecholamines, on enteric pathogens (Lyte, 2016a). Microbial endocrinology represents the union of the fields of microbiology and neurobiology and, as such, views the microbiota as capable of both responding to, as well as producing, neurochemicals that can influence other members of the microbiota as well as the host (Lyte, Vulchanova & Brown, 2011; Freestone, 2013). Microbial endocrinology-based research has shown that the stress-related catecholamines NE and DA can significantly enhance the growth and virulence of enteric pathogens such as *Escherichia coli* O157:H7, *Salmonella enterica*, and *Listeria monocytogenes* (Lyte, Vulchanova & Brown, 2011; Freestone, 2013). Similarly, adding NE or epinephrine (EPI) into iron-restricted media also resulted in increased growth and enhanced virulence of *C. jejuni* NCTC 11168 (Cogan et al., 2007; Xu et al., 2015). As iron is well-recognized to be crucial for the growth of *C. jejuni* (Stahl, Butcher & Stintzi, 2012), it should not be surprising that the catechol moiety found in neurotransmitters such as NE, EPI, and DA, is able to release lactoferrin- and transferrin-bound iron (Lf/Tf-iron) thus making it accessible to bacteria (Sandrini et al., 2010). Although a common mechanism of releasing Lf/Tf-iron is shared by these neurochemicals, individual catecholamines may not act in a uniform manner solely based on iron binding, but may in fact differentially influence bacterial physiology. Transcriptomic analysis of *C. jejuni* cultures treated with NE or EPI demonstrated differentially expressed genes unique to each stress catecholamine treatment (Xu et al., 2015). Thus, increased iron availability is unlikely to be the only way *C. jejuni* may benefit from exposure to NE and DA in intestinal environments.

Critically, while DA is an abundant catecholamine in both small and large intestine (Asano et al., 2012; Sudo, 2019; Eisenhofer et al., 1997), its effect on the growth of *C. jejuni* is unknown. Thus, the present study tested the effect of DA on *C. jejuni* growth in a similar iron-restricted medium system that had been previously used to examine the effect of NE and EPI on *C. jejuni* (Cogan et al., 2007; Xu et al., 2015). By comparing the effect of DA and NE on growth of three *C. jejuni* strains, we demonstrated that the enhanced growth response is dependent on both the type of catecholamine as well as the bacterial strain. Moreover, we identified a new mechanism involving pyruvate, which was shown to modify the response of *C. jejuni* to DA but not NE. Our data suggest that the mechanism of NE- and DA-enhanced growth in *C. jejuni* may be different in a way that the latter requires a physiologically relevant factor, pyruvate, to be present.

## Materials and Methods

### Bacterial strains

Three *C. jejuni* strains, NCTC 11168, 81–176, and ML2126, were used in the study. Strains NCTC 11168 and 81–176 were generously provided by Dr. Matthew Sylte (USDA-ARS, Ames, IA, USA). Strain NCTC 11168 was originally isolated from human diarrheal feces as previously described (Skirrow, 1977). Strain 81–176 was isolated from the stool of a colitis patient as previously reported (Korlath et al., 1985). Both strains NCTC 11168 and 81–176 are well-characterized *C. jejuni* human isolates that are commonly used in animal infection studies to study mechanisms related to pathogenesis (Hofreuter et al., 2006; Pascoe et al., 2019). Strain ML2126 (kindly provided by Dr. Qijing Zhang, Iowa State University) was isolated from the cecal contents of a healthy chicken and identified using MALDI-TOF (Bruker, Billerica, MA, USA). All strains were stored at −80 °C in Muller Hinton (MH) broth (BD, Sparks, MD, USA) supplemented with 20% (v/v) glycerol.

### Chemicals and media

Dopamine hydrochloride (DA) was obtained from AlfaAesar (Tewksbury, MA, USA). L-Norepinephrine bitartrate (NE) was obtained from TCI (Portland, OR, USA). Adult bovine serum, sodium pyruvate, sodium metabisulfite, L-glutathione reduced (GSH), and ferrous sulfate heptahydrate were obtained from Sigma (St. Louis, MO, USA). Adult bovine serum was stored at −20 °C. Prior to inclusion into culture medium , the serum was thawed at 4 °C overnight and sterilized by passing through a 0.2 µm syringe filter.

The *C. jejuni* minimal medium, MCLMAN (Medium Cysteine Leucine Methionine Aspartic acid Niacinamide), was prepared as described (Alazzam et al., 2011). Mueller Hinton broth (MH; Difco, Sparks, MD, USA) and peptone water (Difco, Sparks, MD, USA) were prepared according to manufacturer’s instructions. Two iron-restricted media were prepared as described in previous publications with the modification of utilizing adult bovine serum in place of fetal bovine serum (Cogan et al., 2007; Xu et al., 2015). Specifically, 10% (v/v) adult bovine serum was added into MH and MCLMAN medium which yielded serum-supplemented MH (MHs) and serum-supplemented MCLMAN (MCLMANs), respectively. Blood agar plates (Trypticase soy agar plates supplemented with 10% sheep blood) were used for culture of *C. jejuni* strains from frozen stocks and for quantitative CFU counts (Remel, San Diego, CA, USA).

### Growth assays

*C. jejuni* strains from frozen stocks were cultured on blood agar plates overnight at 41 °C in a microaerophilic atmosphere containing jar (Anoxomat Mark II, Advanced Instruments, Norwood, MA, USA) containing 6% *O*_2_, 7.1% *H*_2_, 7.1% *CO*_2_, and 79.8% *N*_2_. A working bacterial solution was prepared by harvesting bacterial colonies using a sterile cotton swab then suspending into 4 ml of sterile peptone water to yield an *OD*_600_ of 0.2. Immediately prior to inoculation of experimental cultures, this bacterial suspension was further diluted 1000-fold into sterile peptone water. In order to achieve an initial inoculum of approximately 10^4^ colony forming unit (CFU)/ml, 40 µl of the diluted *C. jejuni* suspension was inoculated into 2 ml of iron-restricted medium. Cultures were grown statically in sterile 5 ml round-bottom polystyrene tubes (Falcon) containing 2 ml of culture medium per tube for 24 h in microaerophilic atmosphere containing jars. Following incubation, the CFU per ml in each culture tube was determined using the drop plate method (Chen, Nace & Irwin, 2003), with minor modifications of plating 25 µl for each dilution instead of 10 µl.

Growth assays were performed with the following objectives: (1) To confirm that serum-supplemented medium is iron-restricted, we examined the effect of iron supplementation on *C. jejuni* strain ML2126 in MHs supplemented with different concentrations of ferrous sulfate heptahydrate (0, 5, 10, 20 and 40 µM). (2) To test the effect of stress catecholamines on *C. jejuni* growth, NE or DA were included at physiologically relevant 100 µM in iron-restricted media (Lyte & Ernst, 1992; Eldrup & Richter, 2000). Both types of the iron-restricted media, MHs and MCLMANs, were supplemented with 1 mM sodium pyruvate, named pMHs and pMCLMANs, respectively. The growth of *C. jejuni* strains 81–176, NCTC 11168, and ML2126 in culture media with and without NE or DA were examined using the growth assay. (3) To test the effect of catecholamine concentration on *C. jejuni* growth, growth assays were performed using *C. jejuni* strains NCTC 11168 and 81–176 in pMHs and pMCLMANs supplemented with the following concentrations of NE or DA: 0 µM (no supplementation), 10, 50, 100, 200 and 500 µM. (4) To test the effect of pyruvate supplementation on the response of *C. jejuni* to catecholamines, growth assays were conducted using *C. jejuni* strain NCTC 11168 in MHs and MCLMANs with and without 1 mM sodium pyruvate supplementation. (5) To examine the possibility of pyruvate functioning as an antioxidant, growth assays were performed using *C. jejuni* strain NCTC 11168 in NE or DA supplemented MCLMANs with and without sodium pyruvate or two other antioxidants, sodium metabisulfite or GSH, each of which was supplemented to achieve a concentration of 2 mM in the iron-restricted medium.

### Statistical analysis

Two-tailed t-tests were performed on growth assay data using Prism v8.1.2 (Graph Pad Software Inc., San Diego, CA, USA).

## Results

### Effect of catecholamine supplementation on *C. jejuni* growth in iron-restricted media

**Figure 1:**
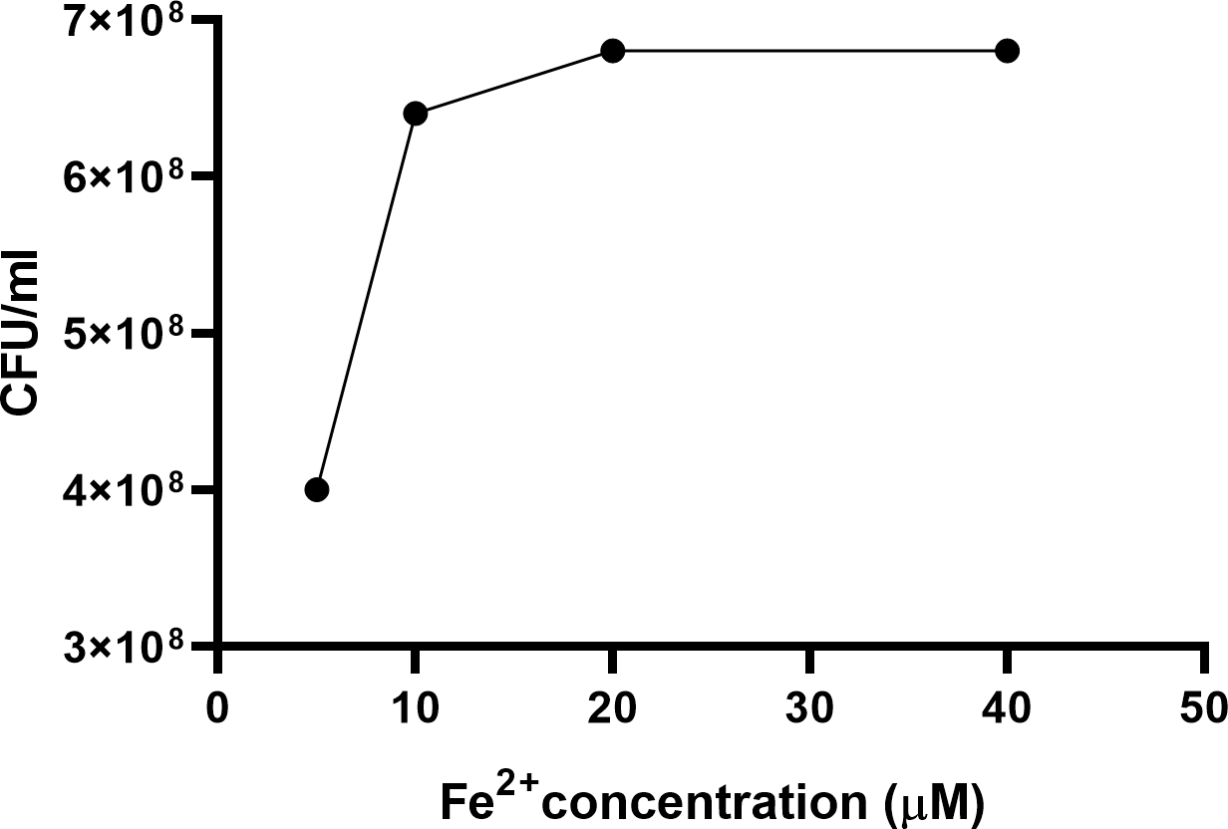
The 24-hour growth of *C. jejuni* ML2126 in MHs supplemented with different concentrations of iron. Data represent the means of duplicate samples.

**Figure 2:**
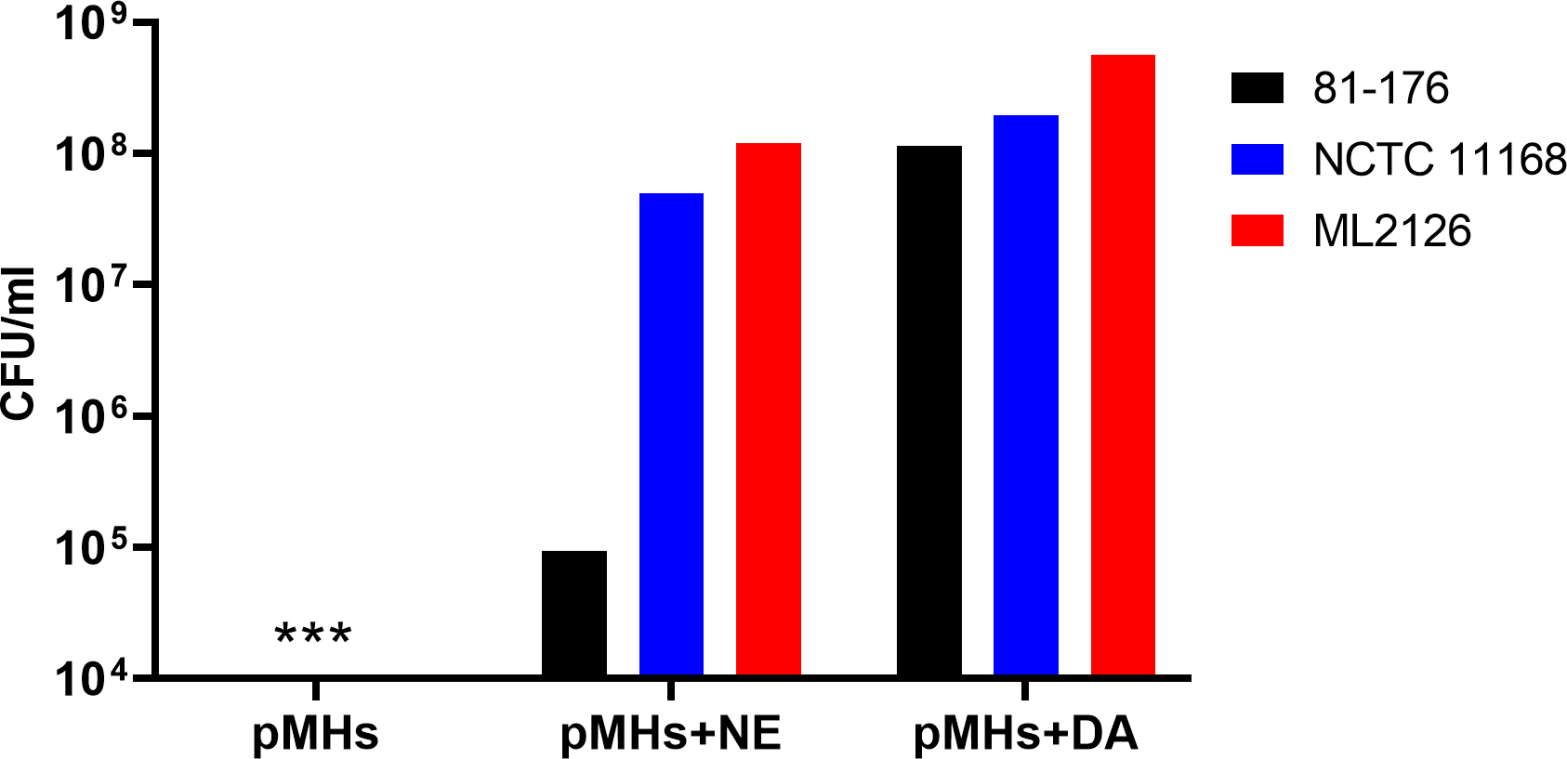
The 24-hour growth of 3 *C. jejuni* strains in pyruvate-supplemented MH broth with serum (pMHs) with and without the supplementation of 100 µM NE or DA. An asterisk (∗) indicates no detectable growth at the end of incubation. Each bar represents the mean of duplicate samples. These data are representative of two independent experiments.

**Figure 3:**
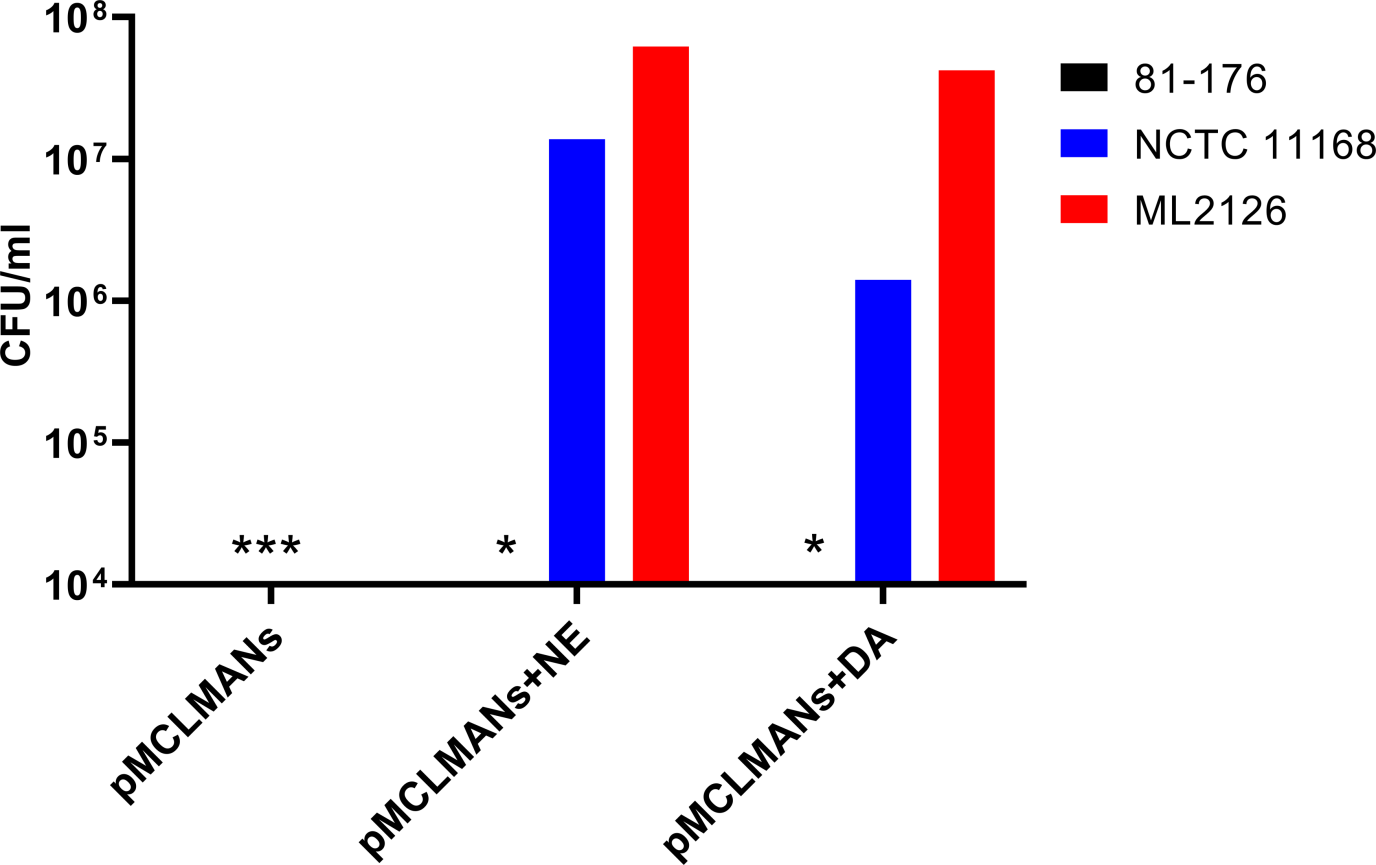
The 24-hour growth of 3 *C. jejuni* strains in pyruvate-supplemented MCLMAN medium with serum (pMCLMANs) with and without the supplementation of DA or NE (100 µM). An asterisk (∗) indicates no detectable growth at the end of incubation. Each bar represents the mean of duplicate samples. The graph is representative of two independent experiments.

Addition of iron in a dose-dependent manner to MHs broth reversed the growth inhibitory effect of the serum component in MHs on *C. jejuni* (Fig. 1). No growth of the three *C. jejuni* strains were detected in the absence of iron. The addition of NE or DA (100 µM) to pyruvate treated iron-restricted medium pMHs enhanced growth of *C. jejuni* strains ML2126 and NCTC 11168 (Fig. 2). Without DA or NE, these strains failed to grow in pMHs (Fig. 2). *C. jejuni* strain 81–176 was unique since DA, but not NE, increased its growth to levels comparable with strains ML2126 and NCTC 11168 grown in pMHs (Fig. 2). Consistent with the result observed in pMHs, in iron-restricted minimal medium pMCLMANs the addition of 100 µM DA or NE also enhanced growth of NCTC 11168 and ML2126 (Fig. 3). *C. jejuni* strain 81–176 failed grow in MCLMAN-based iron-restricted media regardless of catecholamine supplementation (Fig. 3), although it was able to grow in regular MCLMAN medium. Without DA or NE, all three strains failed to grow in pMCLMANs (Fig. 3).

**Figure 4:**
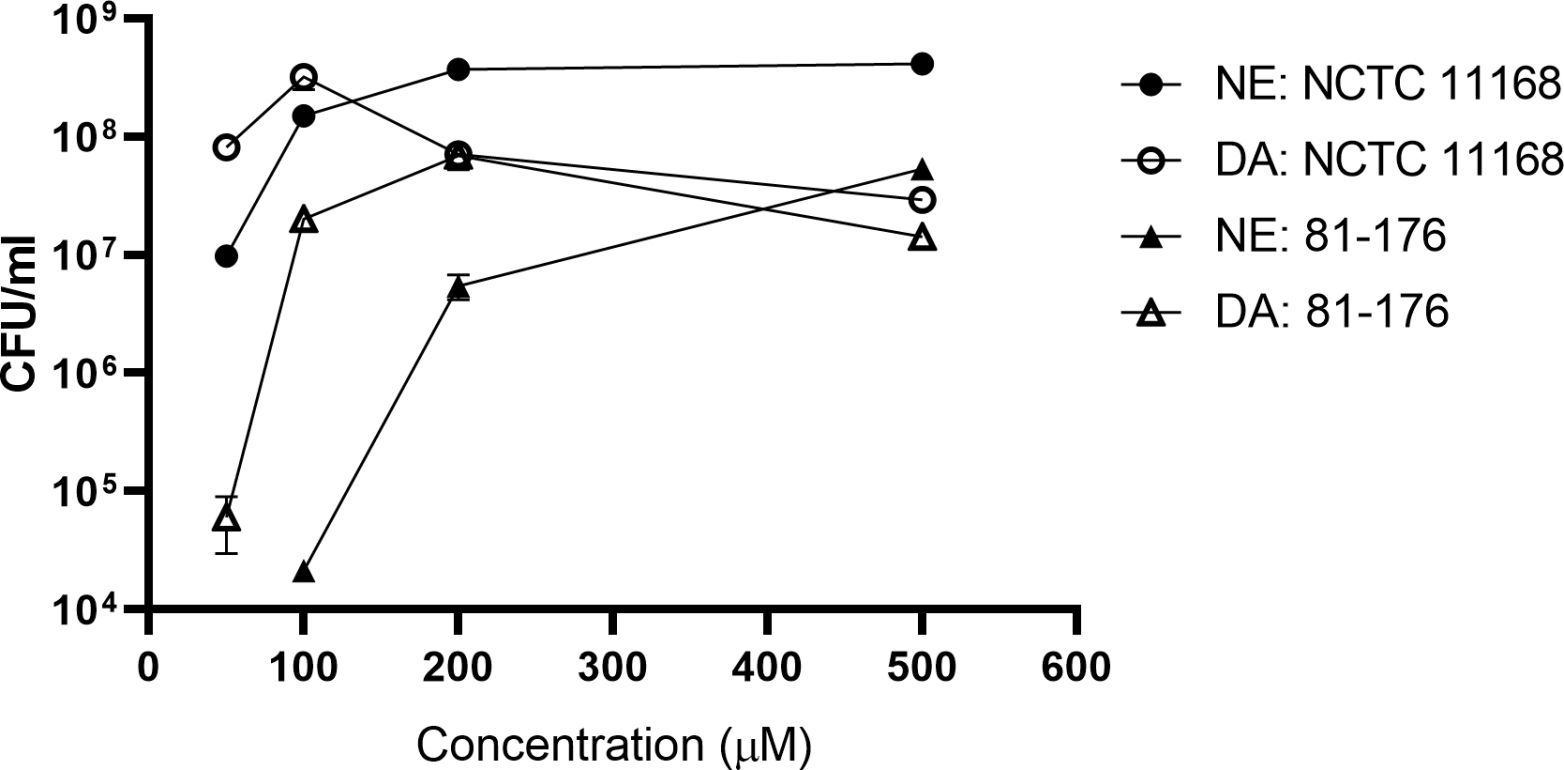
The 24-hour growth of *C. jejuni* NCTC 11168 and 81-176 in pMHs supplemented with different concentrations DA or NE. Each point represents the mean ± standard deviation of triplicate samples.

No growth was observed in pMHs at the end of the 24-hour incubation when the NE or DA concentration was less than 10 µM (Fig. 4). The growth of strains 81–176 and NCTC 11168 were enhanced, respectively, by addition of NE at >100 µM or DA at >50 µM and addition of NE or DA at ≥50 µM (Fig. 4).

### Effect of pyruvate on the response of *C. jejuni* to catecholamines

**Figure 5:**
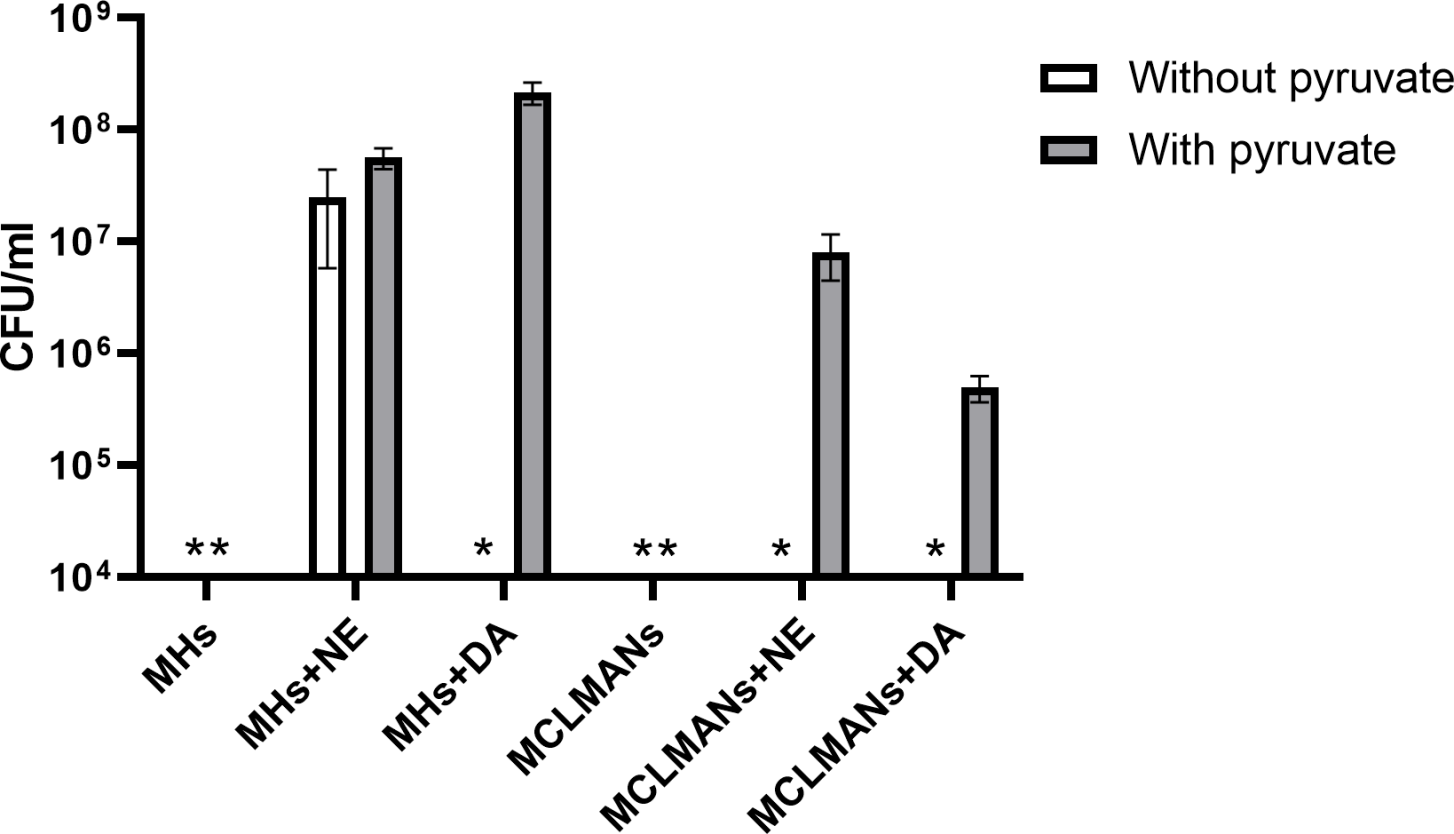
The 24-hour growth of *C. jejuni* strain NCTC 11168 in DA or NE supplemented (100 µM) iron-restricted media MHs or MCLMANs with and without 1 mM sodium pyruvate supplementation. An asterisk (∗) indicates no detectable growth at the end of incubation. Each bar represents the mean ± standard deviation of triplicate samples.

**Figure 6:**
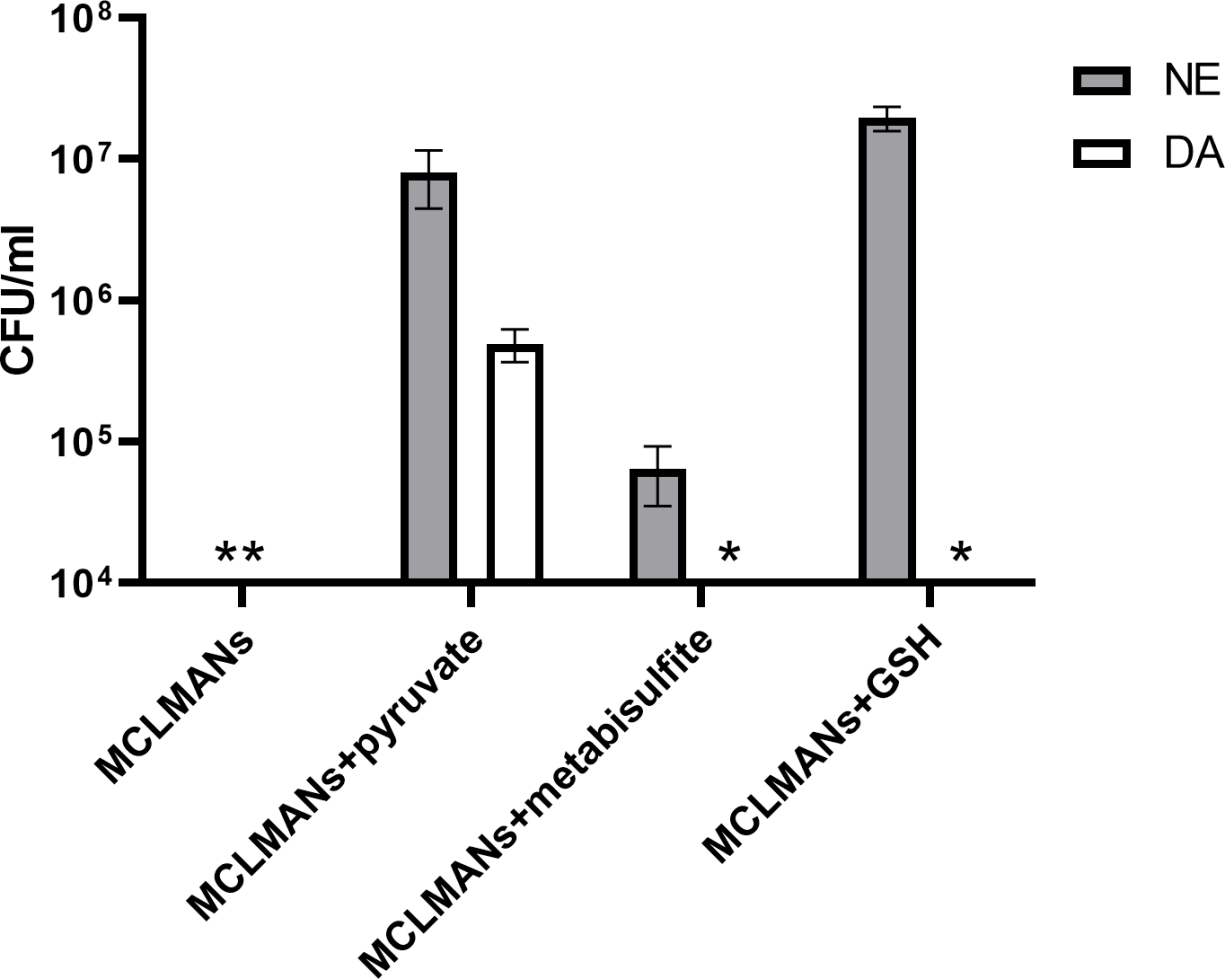
The 24-hour growth of *C. jejuni* NCTC 11168 in DA or NE supplemented (100 µM) MCLMANs with and without different antioxidants included at a concentration of 2 mM. An asterisk (∗) indicates no detectable growth at the end of incubation. Each bar represents the mean ± the standard deviation of triplicate samples.

It has been proposed that the addition of catecholamines may induce oxidative stress in *C. jejuni* strain NCTC 11168 (Xu et al., 2015). Prior work has shown that pyruvate may protect bacteria from oxidative stress (Thompson, Mefferd Jr & Wyss, 1951). Since pyruvate is generated in large amounts in the intestine as a bacterial glycolysis product (Morita et al., 2019; Wu et al., 2014), supplementing it into culture media is also physiologically relevant. To define a mechanism by which pyruvate affects DA or NE-enhanced growth, the growth of strain NCTC 11168 with and without pyruvate addition was examined in rich (MHs) and minimal (MCLMANs) media. Without catecholamine supplementation, *C. jejuni* NCTC 11168 failed to grow in both iron-restricted media whether or not pyruvate was present (Fig. 5). Addition of DA required pyruvate to enhance growth of strain NCTC 11168 in rich or minimal iron-restricted media (Fig. 5). In the absence of pyruvate, addition of NE only slightly enhanced growth of strain NCTC 11168 in the rich medium but not in the minimal medium. Growth in the latter medium was rescued by pyruvate addition (*p* < 0.05) (Fig. 5). Similar effects of pyruvate on DA or NE-enhanced growth were also seen for strain ML2126 (data not shown). Replacing pyruvate with other antioxidants such as GSH or sodium metabisulfite enhanced growth of *C. jejuni* strain NCTC 11168 in pMCLMANs when NE was supplemented (*p* < 0.05), but not when DA was added (Fig. 6).

## Discussion

Microbial endocrinology is an emerging field of research that studies the bidirectional communication between host and microbiota in which neurochemicals serve as a common language shared between both (Lyte, 2016b). As an interdisciplinary research area, the theoretical framework can be employed to examine mechanisms that may be involved in the ability of stress to influence the pathogenesis of infectious disease (Lyte, 2016b; Lyte, 2019). In the present study, we employed a microbial endocrinology-based approach to examine the effect(s) of stress-related catecholamines on *C. jejuni* growth. Studies examining the effects of stress on bacterial physiology through direct, non-immune mediated interactions between host neurophysiology and bacteria are critical to our understanding of infectious disease pathogenesis since both human and food-producing animals unavoidably experience stress. Numerous studies have shown that stress exposure increased susceptibility to bacterial infections (Mawdsley & Rampton, 2005; Franzosa et al., 2019; Zijlmans et al., 2015; Shi et al., 2019; Chen et al., 2018). The present study used a microbial-endocrinology approach to examine the effect(s) of two members of the catecholamine family that are produced within the intestinal tract by the ENS and found in the gut lumen, NE and DA, on the growth of the leading foodborne bacteria, *C. jejuni*. The results shown in the present study extend our understanding of the role of host neurochemicals in the pathogenesis of bacterial disease.

To survive and successfully replicate in the intestinal tract, *C. jejuni* needs to adapt to restricted access to essential nutrients (Tuma & Hubbard, 2003; Sherman et al., 2018; Legrand & Mazurier, 2010). In the intestine, iron is one of the key limiting nutrients making the environment restrictive for *C. jejuni* growth (Stahl, Butcher & Stintzi, 2012). Apart from active iron uptake by small intestine enterocytes (Tuma & Hubbard, 2003; Sherman et al., 2018), the host can also produce high affinity iron-sequestering proteins such as lactoferrin and transferrin, which serves as a primary innate mechanism to limit some bacterial infections. Lactoferrin is secreted from exocrine glands located in the proximal digestive system (Legrand & Mazurier, 2010) and can be found in mucosal secretions such as saliva, bile, pancreatic juice, and gastric fluids (Legrand & Mazurier, 2010; Larkins, 2005). In contrast, transferrin is mainly present in serum and is responsible for delivering iron to host cells through receptor-mediated endocytosis (Enns, Rutledge & Williams, 1996). To simulate the iron-restricted environment bacteria encountered in the gastrointestinal tract, serum-supplemented medium has been utilized in prior studies to examine the effect of catecholamines on enteric pathogenic bacteria (Lyte & Ernst, 1992; Freestone et al., 2000). In the present study, chemically defined and complex serum-containing media were developed based on a medium previously used to examine the response of *C. jejuni* strain NCTC 11168 to NE and EPI (Cogan et al., 2007; Xu et al., 2015). In the present study, no growth of the three *C. jejuni* strains were detected in the serum-containing media (Figs. 2 and 3), whereas supplementing the serum-containing medium with iron rescued the growth of *C. jejuni* (Fig. 1). These data suggest that the growth inhibitory effect of serum-containing media used throughout this study is largely due to iron-restriction. These data are consistent with previous studies which demonstrated iron rescued the growth of enteric pathogenic bacteria such as enteroaggregative *E. coli* (Burton et al., 2002), *Salmonella enterica* (Pullinger et al., 2010), and *Vibrio cholerae* (Halang et al., 2015) in serum-containing medium.

Within the intestinal tract, *C. jejuni* is also exposed to a variety of molecules that are either secreted by the host or the microbiota. One group of these molecules is the catecholamine family, especially NE and DA, which are secreted into the intestinal lumen from the ENS nerve terminals during periods of stress (Lyte & Bailey, 1997; Freestone, 2013; Li et al., 2004; Zhang et al., 2012). These catecholamines may also be provided by other members of the gut microbiota such as *Enterococcus faecium*, *E. coli*, or *Clostridium* spp. whether generated from catecholamine precursors or glucuronide-conjugated catecholamines (Asano et al., 2012; Villageliú & Lyte, 2018). Stress-related catecholamines such as NE and DA enhance both the growth and the virulence of a variety of bacteria in iron-restricted media (Lyte, Vulchanova & Brown, 2011; Freestone, 2013). Catecholamines remove iron from host iron-binding proteins such as lactoferrin and transferrin, through which the iron becomes more accessible to bacteria (Freestone et al., 2000). However, the catecholamine-mediated effects on bacteria may also involve other unknown mechanisms. In the present study, the inclusion of NE into iron-restricted MH or MCLMAN medium significantly enhanced the growth of *C. jejuni* strains NCTC 11168 and ML2126 (Figs. 2 and 3), which is in consistent with the findings in previous studies (Cogan et al., 2007; Xu et al., 2015). To test whether the NE-enhanced growth is present in a more physiologically relevant culture medium, the growth assay was also conducted in an iron-restricted simulated small intestinal medium, since the survival and proliferation of *C. jejuni* in the small intestine is necessary for its colonization. The simulated small intestinal medium (sSIM) was prepared as described Villageliú & Lyte (2018), followed by adding 10% (v/v) adult bovine serum into the medium to restrict iron. Similar to the results obtain in iron-restricted MH and MCLMAN media, in the iron-restricted sSIM, the addition of 100 µM NE also significantly enhanced the growth of *C. jejuni* (data not shown). Dopamine is another stress catecholamine found in high concentrations in the intestine (Asano et al., 2012; Sudo, 2019; Eisenhofer et al., 1997), however, its effect on *C. jejuni* is unknown. Our results suggest that DA enhanced *C. jejuni* growth in iron-restricted medium is similar to that of NE (Figs. 2 and 3). However, the mechanism of the DA-enhanced growth may not be the same as the NE-enhanced growth, which is supported by the observation that pyruvate was a requisite for the DA-enhanced, but not the NE-enhanced, growth (Figs. 5 and 6). It should be noted that the varied response to different catecholamines has been previously shown in a *C. jejuni* (Xu et al., 2015) which demonstrated 30% of differentially expressed genes were due to NE or EPI treatment.

Critically, results from the present study suggest a different mechanism by which *C. jejuni* responds to DA and pyruvate. To our knowledge, this mechanism has not been described previously. Our initial experiments using serum supplemented MCLMAN (MCLMANs) completely failed to demonstrate the NE-stimulated growth in *C. jejuni*, which is in contrast with a previous study where a tissue-based culture medium, *α*MEM, was used instead (Xu et al., 2015). Comparing the formulation of MCLMAN and *α*MEM, we suspected that sodium pyruvate, which is present in *α*MEM but not in MCLMAN medium, may be responsible for this disparity. Supplementing MCLMANs medium with the same level of sodium pyruvate found in *α*MEM medium successfully reproduced the NE-enhanced growth observed in the previous study, suggesting that pyruvate is an important factor in the catecholamine-enhanced growth among *C. jejuni* strains. Catecholamine neurotransmitters such as NE and DA are known to generate reactive oxygen species (ROS) that are harmful to bacteria (Bindoli, Rigobello & Deeble, 1992; Giunta et al., 1990; Guita, Galeazzi & Groppa, 1991; Lyte & Ernst, 1992). Since pyruvate has been previously shown to protect bacteria against the lethal effect of ROS (Thompson, Mefferd Jr & Wyss, 1951), we examined the possibility that pyruvate is acting as an antioxidant. To test this hypothesis, we performed a preliminary experiment in MCLMAN and MH without serum supplementation to which NE or DA was added at a final concentration of 100 µM to the medium. Addition of either catecholamine at 100 µM concentration prevented growth of *C. jejuni* in MCLMAN medium, suggesting that *C. jejuni* is sensitive to ROS generated by the catecholamines. In MH, a significantly lower CFU/ml was also observed in catecholamine-supplemented cultures. Critically, when antioxidants such as sodium pyruvate, sodium metabisulfite and GSH were included into MCLMAN or MH, the growth inhibitory effect of catecholamines were reversed (data not shown). These data are consistent with previous studies with *Staphylococcus aureus*, where the toxicity of catecholamines was also blocked by addition of antioxidants (Giunta et al., 1990; Guita, Galeazzi & Groppa, 1991). Following testing in growth media without serum supplementation, the above antioxidants were then examined in the iron-restricted medium, pMCLMANs. Interestingly, although the inclusion of all 3 antioxidants enabled strain NCTC 11168 to positively respond to NE supplementation as reflected by increased growth, a similar result was not observed with DA supplementation, where only pyruvate supplementation was effective (Fig. 6). Since, pyruvate, a TCA cycle intermediate, is also a recognized energy source, we further tested other energy sources that are known to be utilized by NCTC 11168, including L-serine, L-glutamic acid potassium salt, and monosodium succinate (Wagley et al., 2014). None of these energy sources were able to replace pyruvate in its ability to induce DA-enhanced growth (data not shown). In aggregate, our findings therefore suggest that an antioxidant, such as pyruvate, metabisulfite, or GSH are required in culture medium supplemented with NE or DA to protect *C. jejuni* against ROS generated by the catecholamines. However, in iron-restricted medium, the DA-enhanced growth can only be seen with pyruvate supplementation but not with the addition of other antioxidants or energy sources. Thus, DA-enhanced growth may involve a different mechanism when compared with the NE-enhanced growth in *C. jejuni* where pyruvate plays an important role. The mechanism(s) by which pyruvate participates in the DA-enhanced growth of *C. jejuni* are not well understood.

What is also evident in the present study is the strain specific response to catecholamines. Although *C. jejuni* strains NCTC 11168 and ML2126 had similar response to NE and DA in both types of iron-restricted media, the response of strain 81–176 to the catecholamines was significantly different from the other two strains (Figs. 2 and 3). The strain specific response to NE and DA is best observed in the concentration curves for *C. jejuni* strains NCTC 11168 and 81–176 (Fig. 4), where NCTC 11168 was more sensitive to NE and DA compared with strain 81–176. These data are consistent with the results in Figs. 2 and 3. The difference is more prominent with their response to NE, where NCTC 11168 reached a population density of around 1 × 10^7^ CFU/ml when NE was supplemented at 50 µM, whereas the same NE concentration failed to initiate growth for 81–176. Another trend revealed by the concentration curves is that both *C. jejuni* strains were more responsive to low concentrations (less than 100 µM) of DA than NE in pMHs, with higher CFU/ml achieved in DA-supplemented media than in NE-supplemented media at the end of the incubation. The results further emphasize that different mechanisms between NE- and DA-enhanced growth may be operative in different strains of *C. jejuni*.

The novel discovery of strain-specific response to catecholamines could assist in elucidating the mechanism by which *C.jejuni* utilizes catecholamine-iron complexes. It has been shown that catecholamine-enhanced growth and NE uptake in *Escherichia coli* O157:H7, *Salmonella enterica* and *Yersinia enterocolitica* can be blocked by adrenergic and dopaminergic receptor antagonists (Freestone, Haigh & Lyte, 2007). However, this type of blockage is not present in *C. jejuni* NCTC 11168 nor can the bacterium internalize NE (Cogan et al., 2007). The different response to catecholamins observed in *C. jejuni* strains NCTC 11168 and 81–176 suggests that they have different iron-uptake mechanisms. Considerable progress has been made on understanding iron acquisition mechanisms in *C. jejuni*. NCTC 11168 has been demonstrated to be able to utilize transferrin- and lactoferrin-bound iron (Tf/Lf-iron) in liquid culture through the proposed receptor CtuA (Miller et al., 2008). However, the ability of *C. jejuni* to utilize Tf/Lf-iron may not be adequate in vivo because all *C. jejuni* strains used in the present study were unable to grow in media containing adult bovine serum (10% v/v). Another mechanism that allows bacteria to access Tf/Lf-iron is through the production of siderosphores, a group of high affinity iron chelators with low molecular weight. For example, members of *Enterobacteriaceae* are well recognized for their ability to produce the siderophore enterobactin (Behnsen & Raffatellu, 2016; Raymond, Dertz & Kim, 2003). Two of the *C. jejuni* strains used in this study, NCTC 11168 and 81–176, are known for their ability to utilize siderophores, such as enterobactin and ferrichrome, produced by other intestinal microorganisms (Field et al., 1986; Zeng, Xu & Lin, 2009; Xu et al., 2010). Previous studies have associated NE-enhanced growth with the enterobactin uptake system, which may be explained by the structural similarity between Fe-Ent and Fe-NE complexes (Sandrini et al., 2010). The NE-enhanced growth in *C. jejuni* is also associated with the ferric enterobactin (FeEnt) receptor, CfrA (Zeng, Xu & Lin, 2009). Thus, a study which utilized a NCTC 11168 CfrA mutant showed significantly impaired NE-enhanced growth compared with the wild-type strain, although the mutation did not completely abolish NE-enhanced growth (Zeng, Xu & Lin, 2009). A similar finding was also seen in *E. coli*, where strains with mutations on FeEnt uptake failed to respond to NE (Burton et al., 2002). The uptake of FeEnt may only be partially responsible for catecholamine-enhanced growth since a *C. jejuni* mutant that is fully incapable of utilizing FeEnt has been shown to have similar NE-enhanced growth compared with the wild-type strain (Xu et al., 2010). Therefore, apart from FeEnt utilization pathway, other unknown mechanisms may also contribute to catecholamine-enhanced growth. Another important component in iron uptake systems associated with catecholamine-enhanced growth is the TonB-ExbB-ExbD energy transduction system (TonB complex). Studies which have utilized *E. coli* and *Bordetella bronchiseptica* have demonstrated that a functional TonB complex is required for the NE-enhanced growth in iron-restricted media containing Tf/Lf-iron (Anderson & Armstrong, 2008; Freestone et al., 2003). In gram-negative bacteria, TonB complexes are known to provide energy for iron uptake mediated by other membrane receptors. For example, in *C. jejuni*, the uptake of FeEnt through outer membrane receptor CfrA and CfrB both require an intact TonB-ExbB-ExbD energy transduction system (Zeng, Xu & Lin, 2013). Unlike *C. jejuni* strain NCTC 11168 which has 3 TonB complexes, strain 81–176 only has a single TonB2/ExbB2/ExbD2 complex (Hofreuter et al., 2006), which may explain its less sensitivity to NE and DA observed in the present study.

## Conclusions

By employing a microbial endocrinology-based approach, the present study has identified novel aspects regarding the mechanism(s) by which stress-related neurochemicals, especially members of the catecholamine family, influence the growth of *C. jejuni* in physiologically relevant media. Our results demonstrated that the intestinal stress-related catecholamines, NE and DA, enhanced the growth of *C. jejuni* in a strain-dependent manner. Furthermore, the DA-enhanced growth in *C. jejuni*, unlike the NE-enhanced growth, required pyruvate as a key factor. The results shown in the present study extend our understanding of the mechanisms by which stress may contribute to the pathogenesis of foodborne bacterial infections.

##  Supplemental Information

## Additional Information and Declarations

### Data Availability

The following information was supplied regarding data availability: Raw data are available in the Supplemental Files.
